# MiR-23a sensitizes nasopharyngeal carcinoma to irradiation by targeting IL-8/Stat3 pathway

**DOI:** 10.18632/oncotarget.5117

**Published:** 2015-06-07

**Authors:** Qu Jia-Quan, Yi Hong-Mei, Ye Xu, Li Li-Na, Zhu Jin-Feng, Xiao Ta, Yuan Li, Li Jiao-Yang, Wang Yuan-Yuan, Feng Juan, He Qiu-Yan, Lu Shan-Shan, Yi Hong, Xiao Zhi-Qiang

**Affiliations:** ^1^ Research Center of Carcinogenesis and Targeted Therapy, Xiangya Hospital, Central South University, Changsha, Hunan, China 2; ^2^ The Higher Educational Key Laboratory for Cancer Proteomics and Translational Medicine of Hunan Province, Xiangya Hospital, Central South University, Changsha, Hunan, China

**Keywords:** nasopharyngeal carcinoma, radioresistance, miR-23a, IL-8, Stat3

## Abstract

Radioresistance poses a major challenge in nasopharyngeal carcinoma (NPC) treatment, but little is known about how miRNA regulates this phenomenon. In this study, we investigated the function and mechanism of miR-23a in NPC radioresistance, one of downregulated miRNAs in the radioresistant NPC cells identified by our previous microarray analysis. We observed that miR-23a was frequently downregulated in the radioresistant NPC tissues, and its decrement correlated with NPC radioresistance and poor patient survival, and was an independent predictor for reduced patient survival. *In vitro* radioresponse assays showed that restoration of miR-23a expression markedly increased NPC cell radiosensitivity. In a mouse model, therapeutic administration of miR-23a agomir dramatically sensitized NPC xenografts to irradiation. Mechanistically, we found that reduced miR-23a promoted NPC cell radioresistance by activating IL-8/Stat3 signaling. Moreover, the levels of IL-8 and phospho-Stat3 were increased in the radioresistance NPC tissues, and negatively associated with miR-23a level. Our data demonstrate that miR-23a is a critical determinant of NPC radioresponse and prognostic predictor for NPC patients, and its decrement enhances NPC radioresistance through activating IL-8/Stat3 signaling, highlighting the therapeutic potential of miR-23a/IL-8/Stat3 signaling axis in NPC radiosensitization.

## INTRODUCTION

Nasopharyngeal carcinoma (NPC) is a malignant tumor originated from nasopharyngeal epithelial cells with a remarkable racial and geographical distribution. It is highly prevalent in Southern China and Southern Asia, and poses a very serious health problem in these areas [1]. Radiotherapy (RT) is the preferred treatment for NPC. Although more accurate tumor localization and better RT techniques have contributed to the improvement in the local control of NPC, a major obstacle to achieve long-term survival is radioresistance [2, 3]. However, the underlying molecular mechanisms of NPC radioresistance remain poorly understood.

MiRNAs (miRs) are believed to play fundamental roles in the human cancers, and have a great potential for the diagnosis and treatment of cancer [4]. Regulation of tumor radiosensitivity via miRs-associated mechanisms has attracted much attention in the recent years [5-8]. Over the past few years, several miRs involving in tumor radioresistance, such as miR-23b [9], miR-95 [10], miR-21 [11], let7 [12], miR-205 [13], miR-210 [14], miR-181a [15], miR-125b [16], and miR-324-3p [17] have been identified.

We previously used microarrays to compare the differences of both miRNA and mRNA expression profiles in the NPC cell lines with different radiosensitivity, and found that miR-23a was downregulated, but IL-8 was upregulated in the radioresistant NPC cells, and confirmed that IL-8 is the direct target gene of miR-23a in NPC cells [18]. It has been showed that overexpression of miR-23a in the hepatocellular carcinoma (HCC) cells significantly potentiates the *in vitro* and *in vivo* antitumor effect of etoposide by inhibition of topoisomerase 1 expression, and miR-23a can serve as a potential target in regulating chemosensitivity of HCC cells [19]. Emerging data also showed that miR-23b, miR-23a’s family member, is downregulated in pancreatic cancer, which promotes tumor radioresistance by increasing autophagy [9]. However, the function and mechanism of miR-23a in tumor radioresistance have not been characterized. The diagnostic and therapeutic values of miR-23a in tumor radioresistance are still unclear.

IL-8 is a proinflammatory cysteine-X-cysteine (CXC) chemokine [20]. As a proinflammatory molecule in tumor microenvironment, IL-8 plays as an important role in tumor growth, metastasis and drug response [21]. Previous studies have shown that IL-8 promotes NPC growth and metastasis via autocrine and paracrine [22, 23]. Increased serum and tissue IL-8 levels are associated with the worse prognosis of NPC patients, and can serve as an independent prognostic factor for overall patient survival [22, 24]. However, it is undetermined whether high IL-8 expression level in NPC cells contributes to tumor radioresistance, leading to worse prognosis.

IL-8 executes its biological functions by activating cellular signaling pathways. The signal transducer and activator of transcription 3 (Stat3) regulates the expression of numerous critical mediators of tumor formation and metastasis, and it plays a role in the tumorigenesis and progression of virtually all malignancies including NPC [25, 26]. It has been reported that activation of Stat3 is associated with NPC radioresistance [27], and Stat3 can serve as a therapeutic target for NPC radiosensitization [28]. Although IL-8 can activate multiple cell signaling pathways, it is unknown whether IL-8 increases NPC radioresistance by activating Stat3.

In this study, we found that miR-23a expression was frequently downregulated in the radioresistant NPC tissues, restoration of miR-23a expression increased NPC radiosensitivity both *in vitro and in vivo*, and reduced miR-23a increased NPC radioesistance by activating IL-8/Stat3 signaling. Our study for the first time shows the role and mechanism of miR-23a in tumor radioresistance, which highlights the radiosensitizing potential of miR-23a/IL-8/Stat3 signaling axis in NPC and perhaps in other cancers.

## RESULTS

### Reduced miR-23a is correlated with NPC radioresistance and worse patient prognosis

**Figure 1:**
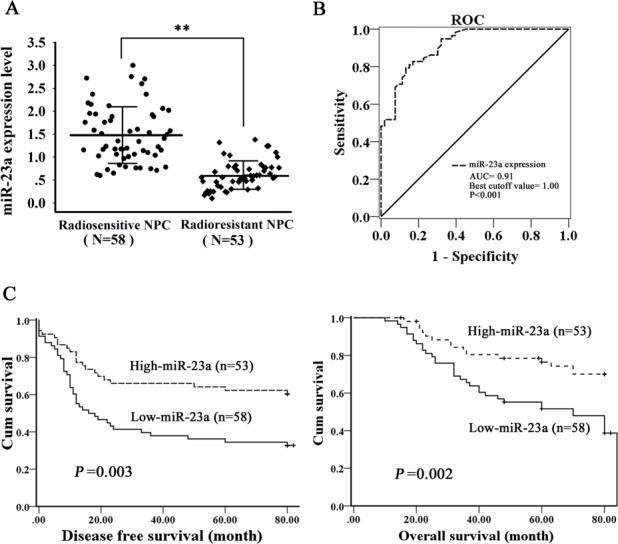
Correlation of miR-23a expression levels with NPC radioresistance and survival of the patients **A.**, qRT-PCR was performed to determine the expression levels of miR-23a in the radioresistant and radiosensitive NPC tissues. Three experiments were done; Means, SDs, and statistical significance are denoted; **, *P* < 0.01. **B.**, receiver-operating characteristic (ROC) analysis was performed to determine the cutoff value of miR-23a that could differentiate between the NPC patients with high and low miR-23a levels (*P* < 0.001; area under the ROC curve, 0.91; cutoff value, 1.00). **C.**, Kaplan-Meier survival analysis for NPC patients according to the expression levels of miR-23a. NPC patients with low miR-23a expression have a significantly worse disease-free survival (left) and overall survival (right) than those with high miR-23a expression. The log-rank test was used to calculate *p* value.

Our previous integrated analysis of differential miRNA and mRNA expression profiles in the radioresistant NPC CNE2-IR and radiosensitive CNE2 cells identified 11 differential miRNAs anticorrelated with mRNA expression (18). We were interested in reduced miR-23a in the CNE2-IR cells, because the function and mechanism of which in tumor radioresistance have not been characterized. Using qRT-PCR, we further detected the levels of miR-23a expression in a cohort of NPC tissues, and found that miR-23a expression was significantly decreased in the radioresistant NPCs relative to radiosensitive NPCs (Figure 1A), and negatively correlated with NPC radioresistance (*r* = −0.715, P < 0.001). The cutoff value of miR-23a determined by receiver-operating characteristic analysis was used to differentiate between the NPC patients with the high and low miR-23a level (Figure 1B). Kaplan-Meier survival analysis for NPC patients was performed based on the expression levels of miR-23a. The results revealed that low miR-23a level in the NPC tissues correlated with the markedly reduced disease-free survival (DFS) and overall survival (OS) of the patients (Figure 1C). A univariate Cox regression analysis showed that miR-23a expression level and clinical TNM stage significantly affected the DFS and OS of NPC patients (Table 1). A multivariate Cox regression analysis confirmed that low miR-23a expression was an independent predictor for the reduced DFS and OS of NPC patients (Table 1). These results indicated the importance of miR-23a expression level in the NPC radioresistance and patient prognosis.

**Table 1:** Univariate and multivariate analyses of prognostic factors for overall and disease-free survival using Cox proportional hazards regression model (*N* =111)

Variable	Disease-free survival				Overall survival			
	Univariate analysis		Multivariate analysis		Univariate analysis		Multivariate analysis	
	HR	95% CI	HR	95% CI	HR	95% CI	HR	95% CI
**Age(y)**								
≥ 46	1		1		1		1	
< 46	0.846	0.504∼1.422	0.784	0.570∼1.442	0.864	0.489∼1.525	0.583	0.534∼1.929
**Gender**								
Male	1		1		1		1	
Female	0.819	0.410∼1.632	0.436	0.320∼1.661	0.980	0.471∼2.041	0.651	0.457∼1.653
**Primary tumor (T)stage**								
T1∼2	1		1		1		1	
T3∼4	0.709	0.411∼1.223	0.841	0.542∼1.347	0.842	0.463∼1.531	0.716	0.638∼1.742
**Lymph node (N) metastasis**								
N0	1		1		1		1	
N1∼3	1.933	0.842∼4.440	1.552	0.803∼3.213	1.740	0.756∼4.006	2.130	0.874∼2.683
**Clinical TNM staging**								
I ∼ II	1		1		1		1	
III ∼ IVa	6.394^$^	2.169∼18.850	6.328^$^	1.261∼17.528	6.191^#^	1.798∼21.319	6.584^#^	2.044∼21.214
**MiR-23a expression level**								
Low	1		1		1		1	
High	0.384^$^	0.222∼0.666	0.435^#^	0.255∼0.743	0.357^$^	0.191∼0.667	0.392^#^	0.214∼0.719

*P < 0.05, #P < 0.01, $P < 0.001. HR, hazard ratio; CI, confidence interval.

### MiR-23a sensitizes NPC cells to irradiation *in vitro*

**Figure 2:**
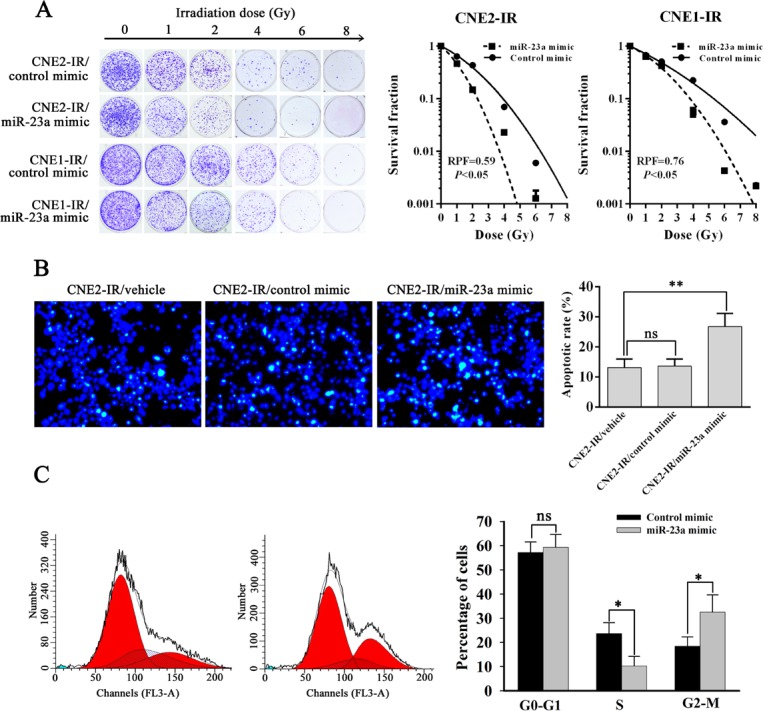
MiR-23a decreases NPC cell radioresistance *in vitro* **A.**, a clonogenic survival assay shows that transfection of miR-23a mimic decreased NPC cell radioresistance compared with transfection of control mimic. (left) CNE2-IR and CNE1-IR cells transiently transfected with control or miR-23a mimic were irradiated with a range of 1-8Gy radiation doses, and colonies that formed after incubation of 12d were stained with crystal violet and photographed; (middle and right) dose survival curves in the CNE2-IR (middle) and CNE1-IR cells (right) transiently transfected with control or miR-23a mimic were created by fitting surviving fractions to the linear quadratic equation. **B.**, Hoechst 33258 staining shows that transfection of miR-23a mimic increased the apoptosis of irradiation-induced CNE2-IR cells compared with transfection of control mimic. (left) CNE2-IR and CNE2-IR cells transiently transfected with control or miR-23a mimic were exposed to 6 Gy irradiation, incubated for 72h, stained with Hoechst 33258 and photographed; (right) a histogram shows the apoptotic rate of CNE2-IR cells and its transfectants. **C.**, a flow cytometry analysis of cell cycle shows that miR-23a mimic-transfected CNE2-IR cells were blocked at G2-M phase by ionizing radiation. (left) a representative result of cell cycle distribution of control or miR-23a mimic-transfected CNE2-IR cells at 24h after 6 Gy irradiation. (right) a histogram shows percentages of cells at each cycle phase in the control or miR-23a mimic-transfected CNE2-IR cells. Three experiments were done; Means, SDs, and statistical significance are denoted; *, *P* < 0.05; **, *P* < 0.05; ns, nonsignificant difference.

To determine the effect of reduced miR-23a on NPC cell radioresistance *in vitro*, CNE2-IR cells were transiently transfected with control or miR-23a mimic, and then cell radiosensitivity was determined. A clonogenic survival assay showed that transfection of miR-23a mimic increased cell radiosensitivity compared with transfection of control mimic [AUC 1.10 (miR-23a mimic) vs. 1.88 (control mimic); *P* < 0.05; RPF = 0.59] (Figure 2A). It is known that irradiation primarily leads to double-strand DNA breaks (DSBs), and unrepaired or misrepaired DSBs in the DNA lead to cell apoptosis. The apoptosis resulting from irradiation is, to a considerable degree, understood as radiosensitivity [29]. Therefore, we also analyzed the effect of miR-23a mimic on the irradiation-induced apoptosis of CNE2-IR cells. Hoechst 33258 staining showed that transfection of miR-23a mimic increased irradiation-induced apoptosis of CNE2-IR cells compared with transfection of control mimic [26.72 &plusmn; 4.43% (miR-23a mimic) vs. 13.61 &plusmn; 2.35% (control mimic); *P* < 0.05] (Figure 2B). Our previous study showed that compared with radiosensitive CNE-2 cells, more CNE-2-IR cells were found detained in S phase with less cells in G2-M phase after 6Gy irradiation [30], which is consistent with the typical radioresistant phenotype [31]. Accordingly, the difference in response to radiation between control and miR-23a mimic transfected-CNE2-IR cells was further studied by cell cycle analysis using flow cytometry. As showed in Figure 2C, no difference was induced by irradiation in G0-G1 phase at 24h after 6Gy ionizing radiation, whereas compared with control mimic-transfected cells, lesser miR-23a mimic-transfected cells were found detained in S phase [10.27 &plusmn; 3.95% (miR-23a mimic) vs. 23.68 &plusmn; 4.49% (control mimic); *P* < 0.05] with more cells in G2-M phase [32.55 7.12% (miR-23a mimic) vs. 18.47 &plusmn; 3.79% (control mimic); *P* < 0.05], suggesting that miR-23a mimic-transfected CNE2-IR cells were blocked at G2-M phase by irradiation, which is consistent with the typical radiosensitive phenotype. Moreover, transfection of miR-23a mimic was also able to increase radiosensitivity in the additional radioresistant NPC CNE1-IR cells [AUC 1.84 (miR-23a mimic) vs. 2.42 (control mimic); *P* < 0.05; RPF = 0.76] (Figure 2A) [32]. Taken together, these results demonstrated that restoration of miR-23a expression could significantly sensitize radioresistant NPC cells to irradiation *in vitro*.

### MiR-23a sensitizes NPC cells to irradiation *in vivo*

**Figure 3:**
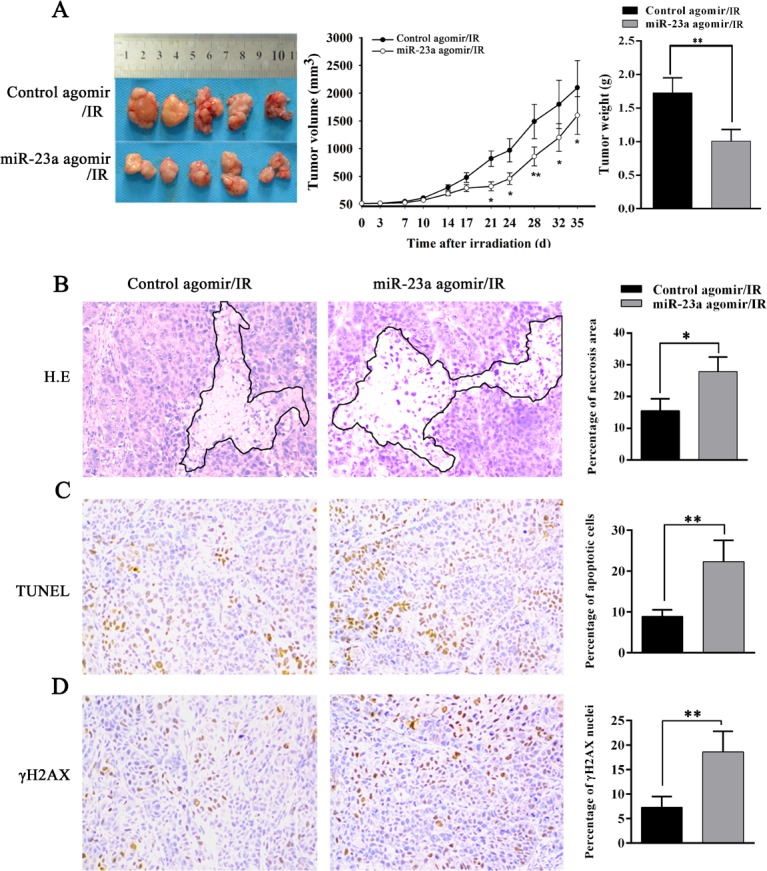
MiR-23a decreases NPC cell radioresistance *in vivo* **A.**, the growth and weight of control or miR-23a agomir-injected CNE2-IR xenograft tumors after irradiation. (left) 5 nmol control or miR-23a agomir was injected into CNE2-IR xenografts before and after 8 Gy ionizing radiation. 5 weeks after irradiation, the mice were killed, and the tumors were photographed; (middle) the growth curves of control or miR-23a agomir-injected CNE2-IR tumors (*n* = 5 each group) at the sacrifice with respect to the first measurements after irradiation; (right) the average weights of control or miR-23a agomir-injected CNE2-IR tumors (*n* = 5 each group) at the sacrifice. **B.**, (left) a representative image of H&E staining of control or miR-23a agomir-injected CNE2-IR tumors with regions of necrosis outlined after irradiation; (right) a histogram shows percentages of necrosis areas in the tumors (*n* = 5 each group). **C.**, (left) a representative image of TUNEL detection of apoptotic cells in the control or miR-23a agomir-injected CNE2-IR tumors after irradiation; (right) a histogram shows percentages of apoptotic cells in the tumors (*n* = 5 each group). **D.**, (left) a representative image of immunohistochemical staining for γH2AX in the control or miR-23a agomir-injected CNE2-IR tumors after irradiation; (right) a histogram shows percentages of γ-H2AX positive cells in the tumors (*n* = 5 each group). Means, SDs, and statistical significance are denoted; *, *P* < 0.05; **, *P* < 0.01. Original magnification, &times;200.

To determine the *in vivo* radiosensitization effect of miR-23a in NPC, we generated subcutaneous tumors in nude mice using radioresistant CNE2-IR cells. Control or miR-23a agomir was injected into the tumors before and after 8Gy ionizing radiation, and radioresponse of xenograft tumors was assessed. As shown in Figure 3A, radiosensitivity of miR-23a-agomir-injected tumors was significantly higher than that of control agomir-injected tumors as demonstrated by tumor growth and weight. H&E staining of tumor tissue sections showed that more necrosis was noted in miR-23a agomir-injected tumors compared with control agomir-injected tumors [27.83 &plusmn; 4.67% (miR-23a-agomir) vs. 15.65 &plusmn; 3.82% (control agomir); *P* < 0.05] (Figure 3B). TUNEL assay showed that more apoptotic cells were present in the miR-23a agomir-injected tumors compared with control agomir-injected tumors [22.35 &plusmn; 5.24% (miR-23a-agomir) vs. 8.92 &plusmn; 1.62% (control agomir); *P* < 0.05] (Figure 3C). Immunohistochemical staining indicated that more positive cells of γH2AX, i.e. more cells with DNA damage, were present in the miR-23a agomir-injected tumors compared with control agomir-injected tumors [18.61 &plusmn; 2.28% (miR-23a-agomir) vs. 7.33 &plusmn; 2.23% (control agomir); *P* < 0.05] (Figure 3D). Taken together, these results demonstrated that restoration of miR-23a expression in NPC cells obviously increased *in vivo* NPC cell radiosensitivity, suggesting that *in vivo* administration of miR-23a had considerable potential for NPC radiosensitization.

### MiR-23a increases NPC cell radiosensitivity through targeting IL-8

**Figure 4:**
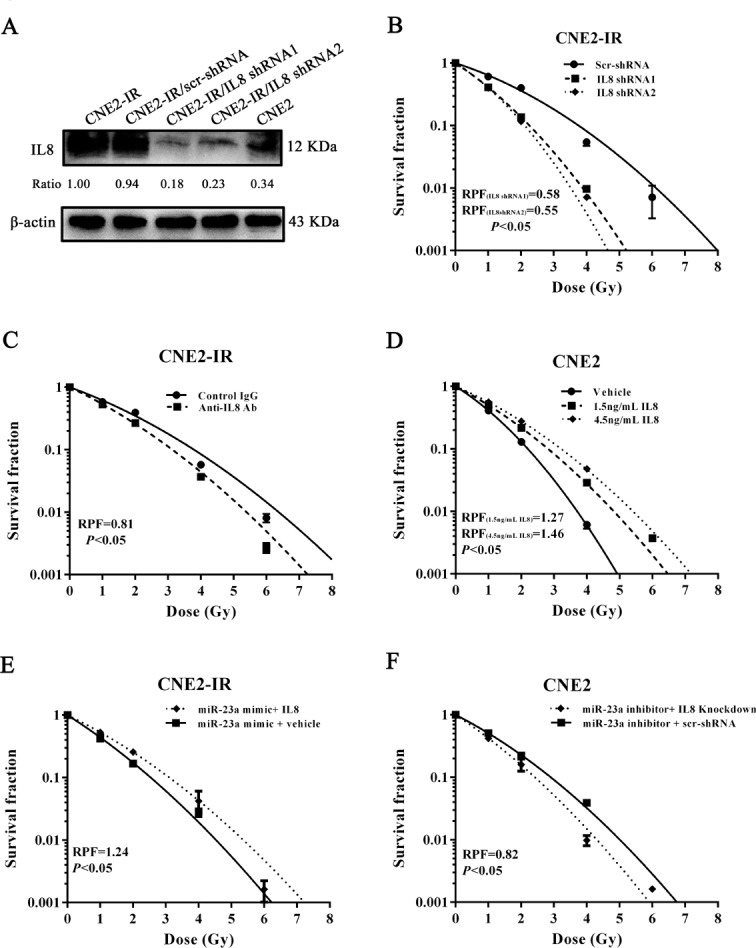
MiR-23a increases NPC cell radiosensitivity through targeting IL-8 **A.**, a representative result of Western blotting shows IL-8 expression levels in CNE2, CNE2-IR, CNE2-IR cells stably transfected with IL-8 shRNA 1, IL-8 shRNA 2 or scramble shRNA vector; **B.**, a clonogenic survival assay shows that knockdown of IL-8 decreased CNE2-IR cell radioresistance. CNE2-IR cells stably transfected with IL-8 shRNA 1, IL-8 shRNA 2 or scramble shRNA vector were irradiated with a range of 1-8Gy radiation doses, and dose survival curves were created by fitting surviving fractions to the linear quadratic equation. **C.**, a clonogenic survival assay shows that antibody neutralization of secretory IL-8 decreased CNE2-IR cell radioresistance. CNE2-IR cells treated with 2.5 mg/mL IL-8 antibody or control IgG were irradiated with a range of 1-8Gy radiation doses, and dose survival curves were created by fitting surviving fractions to the linear quadratic equation. **D.**, a clonogenic survival assay shows that exogenous IL-8 stimulation increased CNE2 cell radioresistance. CNE2 cells stimulated with 1.5, 4.5 ng/mL IL-8 or vehicle were irradiated with a range of 1-8Gy radiation doses, and dose survival curves were created by fitting surviving fractions to the linear quadratic equation. **E.**, a clonogenic survival assay shows that exogenous IL-8 stimulation significantly abolished the radiosensitizing effect of miR-23a mimic in the radioresistant CNE2-IR cells. MiR-23a mimic-transfected CNE2-IR cells treated with 4.5 ng/mL IL-8 or vehicle were irradiated with a range of 1-8Gy radiation doses, and dose survival curves were created by fitting surviving fractions to the linear quadratic equation. **F.**, a clonogenic survival assay shows that IL-8 knockdown markedly abolished radioresistance induced by transfection of miR-23a inhibitor in the radiosensitive CNE2 cells. CNE2 cells transiently cotransfected with miR-23a inhibitor and IL-8 shRNA 1 or scramble shRNA vector were irradiated with a range of 1-8Gy radiation doses, and dose survival curves were created by fitting surviving fractions to the linear quadratic equation. Means, SDs, and statistical significance are denoted.

**Figure 5:**
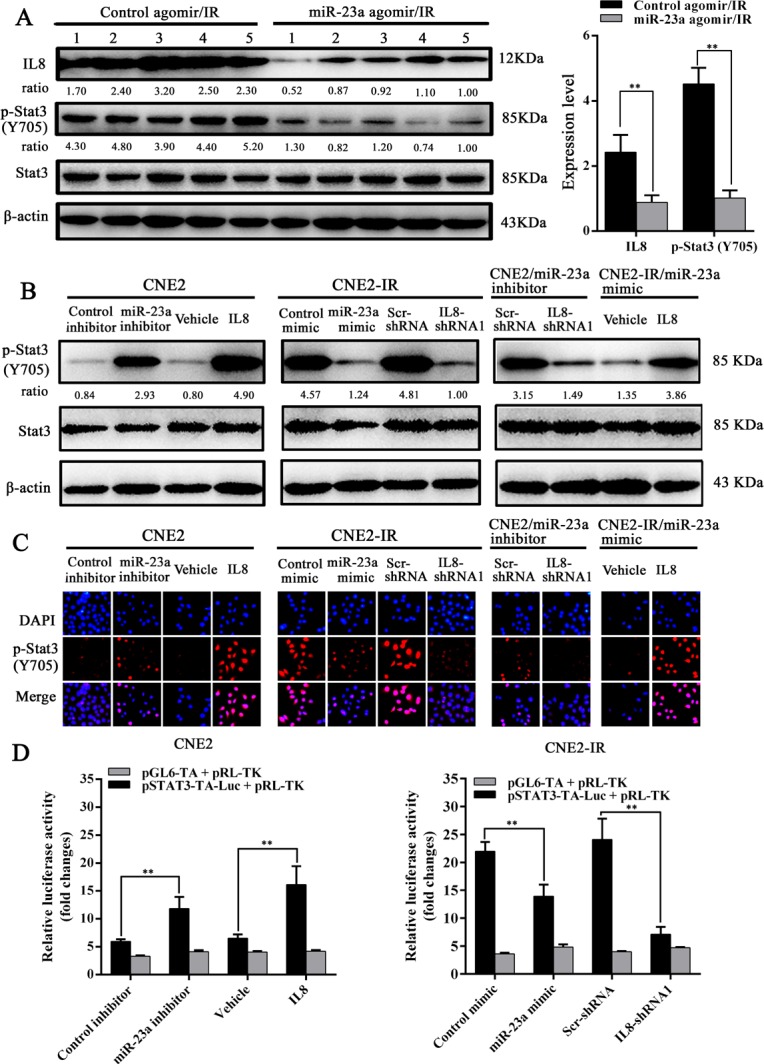
MiR-23a inhibits Stat3 activity by targeting IL-8 in NPC cells **A.**, (left) a representative result of Western blotting analysis shows the expression levels of IL-8 and phosphor-Stat3 in the control or miR-23a agomir-injected CNE2-IR xenografts; (right) a histogram shows the average levels of IL-8 and phosphor-Stat3 in the tumors (*n* = 5 each group). **B.**, a representative result of Western blotting analysis shows phospho-Stat3 levels in the IL-8-stimulated, miR-23a inhibitor-transfected, or miR-23a inhibitor and IL-8 shRNA 1-cotransfected CNE2 cells, and IL-8 knockdown, miR-23a mimic-transfected or miR-23a mimic-transfected and IL-8-stimulated CNE2-IR cells as well as their corresponding controls. **C.**, a representative result of immunofluorescent staining shows the nuclear translocation of phospho-Stat3 in the IL-8-stimulated, miR-23a inhibitor-transfected, or miR-23a inhibitor and IL-8 shRNA 1-cotransfected CNE2 cells, and IL-8 knockdown, miR-23a mimic-transfected or miR-23a mimic-transfected and IL-8-stimulated CNE2-IR cells as well as their corresponding controls. **D.**, Stat3 luciferase reporter activity in the miR-23a inhibitor-transfected or IL-8-stimulated CNE2 cells, and miR-23a mimic-transfected or IL-8 knockdown CNE2-IR cells. Means, SDs, and statistical significance are denoted; **, *P* < 0.01.

Our previous study has confirmed that IL-8 is the direct target of miR-23a in NPC cells [18]. As IL-8’s contribution in tumor radioresistance remains elusive, we further investigated whether IL-8 mediates miR-23a-regulated NPC radioresponse. CNE2-IR cell lines with stable knockdown of IL-8 by IL-8 shRNA and control cell lines transfected with scramble non-target shRNA were established (Figure 4A, Supplementary Figure 1), and used to analyze the effects of IL-8 knockdown on cell radioresponse. A clone survival assay showed that IL-8 knockdown increased cell radiosensitivity [AUC 1.03(IL-8 shRNA 1) vs.1.72 (scramble shRNA), *P* < 0.05; RPF = 0.58] [AUC 0.98 (IL-8 shRNA 2) vs.1.72 (scramble shRNA), *P* < 0.05; RPF = 0.55] (Figure 4B), phenocopying that seen in the miR-23a mimic-transfected cells. Moreover, neutralization of secretory IL-8 using anti-IL-8 antibody increased CNE2-IR cell radiosensitivity compared with control IgG [AUC 1.42 (IL-8 antibody) vs.1.74 (control IgG), *P* < 0.05; RPF = 0.81] (Figure 4C). We also investigated whether exogenous IL-8 stimulation decreases NPC cell radiosensitivity, and found that exogenous IL-8 stimulation markedly decreased radiosensitivity of radiosensitive CNE-2 cells as demonstrated by a clone survival assay [AUC1.28 (1.5 ng/mL IL-8) vs.1.01(control), *P* < 0.05; RPF = 1.27][AUC 1.48 (4.5 ng/mL IL-8) vs.1.01(control), *P* < 0.05; RPF = 1.46] (Figure 4D). Importantly, exogenous IL-8 stimulation markedly abolished the radiosensitizing effect of miR-23a mimic in the radioresistant CNE2-IR cells [AUC 1.41 (IL-8 plus miR-23a mimic) vs. 1.13 (miR-23a mimic), *P* < 0.05; RPF = 1.24] (Figure 4E), whereas IL-8 knockdown markedly abolished radioresistance induced by transfection of miR-23a inhibitor in the radiosensitive CNE2 cells [AUC (IL-8 shRNA1 plus miR-23a inhibitor) vs. (scramble shRNA plus miR-23a inhibitor), *P* < 0.05; RPF = 0.82] (Figure 4F). Moreover, IL-8 level was significantly downregulated in the miR-23a agomir-injected tumors compared with control agomir-injected tumors (Figure 5A), supporting our *in vitro* results. Taken together, our results demonstrated that upregulation of miR-23a increased NPC cell radiosensitivity through targeting IL-8.

### Stat3 signaling mediates miR-23a/IL-8-regulated NPC cell radioresponse

**Figure 6:**
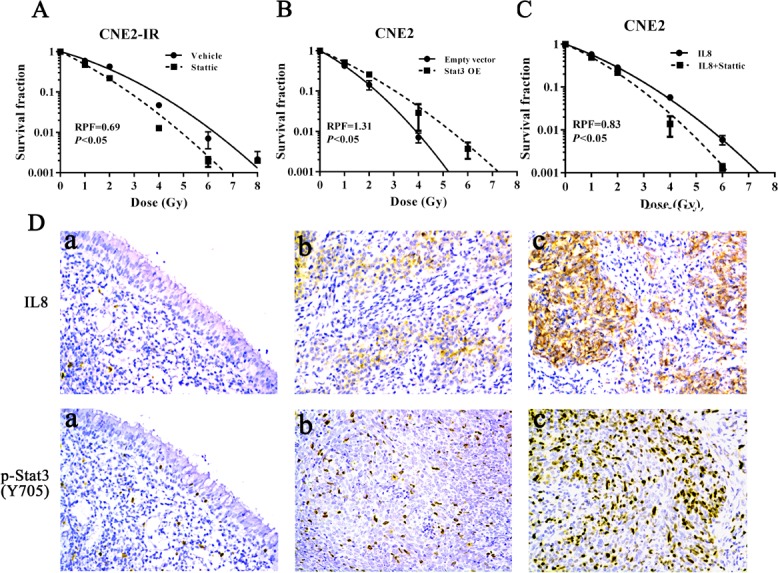
Stat3 signaling mediates miR-23a/IL-8-regulated NPC cell radioresponse **A.**, a clonogenic survival assay shows that inhibition of Stat3 activity decreased CNE2-IR cell radioresistance. CNE2-IR cells treated with 5 &micro;mol/L Stattic or vehicle were irradiated with a range of 1-8Gy radiation doses, and dose survival curves were created by fitting surviving fractions to the linear quadratic equation. **B.**, a clonogenic survival assay shows that Stat3 overexpression (OE) increased CNE2 cell radioresistance. CNE2-IR cells transfected with 4 μg/mL Stat3 expression or control vector were irradiated with a range of 1-8Gy radiation doses, and dose survival curves were created by fitting surviving fractions to the linear quadratic equation. **C.**, a clonogenic survival assay shows that inhibition of Stat3 signaling significantly abolished CNE2 cell radioresistance induced by exogenous IL-8 stimulation. CNE2 cells treated with 5 &micro;mol/L Stattic and 4.5 ng/mL IL-8 were irradiated with a range of 1-8Gy radiation doses, and dose survival curves were created by fitting surviving fractions to the linear quadratic equation. **D.**, a representative image of IL-8 and phospho-Stat3 immunohistochemical staining in the normal nasopharyngeal mucosal tissue (a), radiosensitive NPC tissue (b) and radioresistant NPC tissue (c). Original magnification, &times;200. Means, SDs, and statistical significance are denoted.

Activation of Stat3 is associated with NPC radioresistance (27, 28). Therefore, we investigated whether Stat3 signaling mediates miR-23a/IL-8-regulated NPC cell radioresponse. Western blotting and immunofluorescence staining showed that either exogenous IL-8 stimulation or transfection of miR-23a inhibitor significantly enhanced the phosphorylated level and nuclear translocation of phospho-Stat3 in the radiosensitive CNE2 cells, whereas either IL-8 knockdown or transfection of miR-23a mimic significantly reduced those of phospho-Stat3 in the radioresistant CNE2-IR cells (Figure 5B and 5C). Importantly, IL-8 knockdown significantly abrogated the increased phosphorylated level and nuclear translocation of phospho-Stat3 induced by transfection of miR-23a inhibitor in the radioresensitive CNE2 cells, whereas exogenous IL-8 stimulation restored the phosphorylated level and nuclear translocation of phospho-Stat3 decreased by transfection of miR-23a mimic in the radioresistant CNE2-IR cells (Figure 5B and 5C). A dual luciferase reporter assay also showed that Stat3 luciferase reporter activity was significantly increased in the miR-23a inhibitor-transfected or IL-8-stimulated CNE2 cells, whereas significantly reduced in the miR-23a mimic-transfected or IL-8 knockdown CNE2-IR cells (Figure 5D). These results indicated that miR-23a inhibited Stat3 activation and transcriptional activity by targeting IL-8. Moreover, a clone survival assay showed that Stat3 inhibitor Stattic treatment increased CNE2-IR cell radiosensitivity [AUC 1.24 (Stattic) vs. 1.79 (vehicle), *P* < 0.05; RPF = 0.69] (Figure 6A), whereas Stat3 overexpression decreased CNE2 cell radiosensitivity [AUC 1.37 (Stat3 overexpression) vs. 1.05 (control vector), *P* < 0.05; RPF = 1.31] (Figure 6B). Importantly, Stattic treatment significantly abolished cell radioresistance induced by exogenous IL-8 stimulation in the radiosensitive CNE2 cells [AUC 1.26 (IL-8 plus Stattic) vs. 1.51 (IL-8), *P* < 0.05; RPF = 0.83] (Figure 6C).

**Figure 7:**
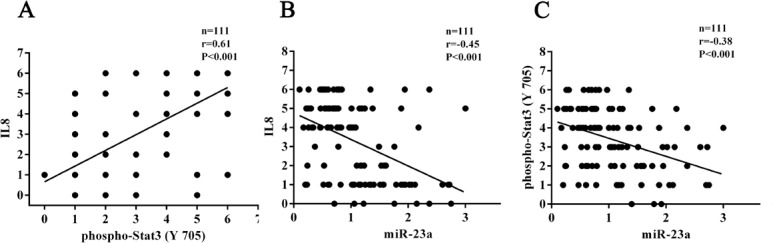
Correlation analyses based on IHC score and qRT-PCR 2-DDCt value (Spearman rank correlation test) The correlations shown include those between IL-8 and phospho-Stat3 **A.**, IL-8 and miR-23a **B.** and phospho-Stat3 and miR-23a **C.**.

In the cohort of NPC tissues, the levels of IL-8 and phospho-Stat3 were significantly higher in the radioresistant NPCs than those in the radiosensitive NPCs (Figure 6D, Table 2). Correlation analyses revealed that IL-8 level was positively associated with phospho-Stat3 level (*r* = 0.61, *P* < 0.001), whereas negatively associated with miR-23a level(*r* = −0.45, *P* < 0.001), and phospho-Stat3 level was negatively associated with miR-23a level (*r* = −0.38, *P* < 0.001) (Figure 7). Moreover, phospho-Stat3 expression was significantly reduced in the miR-23a agomir-injected tumors relative to the control agomir-injected tumors (Figure 5A). Taken together, these results suggested that Stat3 signaling mediated miR-23a/ IL-8-regulated NPC radioresponse, and reduced miR-23a increased NPC radioresistance by activating IL-8/Stat3 signaling.

**Table 2:** The levels of IL-8 and phospho-Stat3 in the nasopharygeal carcinoma and normal nasopharyngeal mucosal tissues

	NNMT	Radiosensitive NPC	Radioresistant NPC
**IL8**			
Low(0-3)	16	40	11
High(4-6)	0	18	42
**Phospho-Stat3**			
Low(0-3)	16	34	18
High(4-6)	0	24	35

## DISCUSSION

Radioresistance is the main obstacle in the current clinical management of NPC [2, 3]. Preventing, predicting, and decreasing NPC radioresistance is therefore critical for further improving the survival rate of patients with NPC. Investigating the role of miRNAs in radioresistance is a promising avenue given their ability to regulate multiple oncogenic processes including response to therapy [8]. We previously performed the integrated analysis of miRNA and mRNA expression profiles in the radioresistant and radiosensitive NPC cell lines, and identified miR-23a as one of downregulated miRNAs in the radioresistant NPC cells [18]. However, the function and mechanism of miR-23a in tumor radioresistance are still unclear.

To gain insight into miR-23a function, we performed *in vitro* and *in vivo* radioresponse tests, and found that upregulation of miR-23a expression markedly increased tumor radiosensitivity in the NPC cells and xenograft tumors, demonstrating that reduced miR-23a enhances NPC radioresistance. These findings are clinically relevant, given our discovery that miR-23a is significantly downregulated in the radioresistant NPC tissues, and its decrement correlated with NPC radioresistance and poor patient survival, outlining a potential marker for predicting the radioresponse and prognosis of NPC patients. To our knowledge, it is for the first time reported that miR-23a modulates tumor radioresponse.

MiRNAs function mainly through inhibition of target gene expression. Our previous study has proved that IL-8 is a direct target of miR-23a in NPC cells [18]. Although IL-8 plays a crucial role in NPC growth [23] and metastasis [24], and increased IL-8 level is associated with worse prognosis of NPC [22, 24], IL-8’s contribution in tumor radioresistance remains elusive. Therefore, we are interesting to investigate whether IL-8 mediates miR-23a-regulated NPC radioresponse. Knockdown of IL-8 or antibody neutralization of secretory IL-8 increased NPC cell radiosensitivity, phenocopying that seen in the miR-23a mimic-transfected NPC cells, whereas exogenous IL-8 stimulation increased NPC cell radioresistance. Furthermore, exogenous IL-8 stimulation abolished the radiosensitizing effect of miR-23a mimic, and IL-8 knockdown abolished radioresistance induced by transfection of miR-23a inhibitor in NPC cells. In the clinical NPC samples, IL-8 level was significantly lower in the radioresistant NPCs than that in the radiosensitive NPCs, and negatively associated with miR-23a level. These results demonstrate that reduced miR-23a enhances NPC radioresistance through targeting IL-8.

MiRNAs modulate tumor radiosensitivity by targeting signal pathways [8]. As activation of Stat3 is associated with NPC radioresistance [27, 28], we investigated whether Stat3 signaling mediates miR-23a/IL-8-regulated NPC cell radioresponse. We observed that transfection of miR-23a inhibitor or exogenous IL-8 stimulation enhanced, whereas transfection of miR-23a mimic or IL-8 knockdown reduced the phosphorylated level and nuclear translocation of Stat3 and its transcriptional activity in NPC cells, indicating that miR-23a inhibited Stat3 activity by targeting IL-8. MiR-23a agomir significantly reduced the levels of IL-8 and phospho-Stat3 in the NPC xenografts, supporting our *in vitro* findings. Moreover, Stat3 inhibitor Stattic not only decreased NPC cell radioresistance, but also markedly abrogated NPC cell radioresistance induced by IL-8 stimulation. In the clinical NPC samples, phospho-Stat3 level was significantly higher in the radioresistance NPCs than that in the radiosensitive NPCs, and positively associated with IL-8 level while negatively associated with miR-23a level. Taken together, our results not only demonstrate that activation of Stat3 by IL-8 enhances NPC radioresistance, but also suggest that reduced miR-23a increases NPC radioresistance by activating IL-8/Sat3 signaling.

As radioresistance is a major cause of treatment failure for NPC, methods for radiosensitization of NPC attract much attention [33-36]. In this study, we confirmed that restoration of miR-23a expression by using miR-23a agomir enhanced NPC radiosensitivity in NPC xenografts, suggesting its considerable potential in radiosensitizing NPC. Nucleic-acid drugs, such as miRNAs, can be directly synthesized and modified to be more lipophilic that improves penetration. Such modification includes cholesterylation. Our delivery of miR-23a agomir, which is cholesterylated miRNA mimic, successfully increased NPC radiosensitivity in the intratumoral injection model, suggesting that miR-23a agomir has a potential for further drug development. Our results also highlight the possibility of radiosensitizing NPC with reduced miR-23a by inhibiting IL-8/Stat3 signaling, given that increased level of IL-8 and phospho-Stat3 was seen in the radioresistant NPCs with reduced miR-23a, and IL-8 knockdown and Stat3 inhibitor Stattic treatment significantly abrogated NPC cell radioresistance.

Although IL-8/Stat3 signaling seems to largely account for the radioresistant phenotype induced by reduced miR-23a, indeed a single miRNA has been thought to target multiple mRNAs to regulate gene expression [37]. Therefore there may be other molecules or signaling pathways which are also targeted by miR-23a [19, 38], and some of them may be still unknown in NPC. This presumption may raise interesting future work to reveal the entire functions of miR-23a in NPC radioresistance.

In summary, our data demonstrate that: i) miR-23a is frequently downregulated in the radioresistant NPC tissues, and its decrement significantly correlates with NPC radioresistance, and is an independent predictor for the poor survival of NPC patients; ii) upregulation of miR-23a increases NPC cell radiosensitivity both *in vitro* and *in vivo*; iii) reduced miR-23a increases NPC radioresistance through activating IL-8/Stat3 signaling. Our study demonstrates that miR-23a is a critical determinant of NPC radioresponse, its expression level in the primary tumor can be used for predicting radioresponse of NPC patients, and targeting miR-23a/IL-8/Stat3 signaling might be a promising approach for enhancing NPC sensitivity to radiotherapy.

## MATERIALS AND METHODS

### Cell lines

Radioresistant human NPC cell lines CNE2-IR and CNE1-IR as well as their corresponding radiosensitive cell lines CNE2 and CNE1 were previously established [30, 32], and were cultured with RPMI-1640 medium containing 10% FBS (Invitrogen, Carlsbad, CA, USA). Radioresistant CNE2-IR and CNE1-IR cells were derived from parental CNE2 and CNE1 cells, respectively, by treating the cells with four rounds of sublethal ionizing radiation [30, 32]. Radiosensitive CNE2 and CNE1, used as a control, were treated with the same procedure except sham irradiated. Experiments were performed with CNE2-IR and CNE1-IR cells within 4 to 10 passages after the termination of irradiation, and their radioresistance was tested by a clonogenic survival assay before use.

### Patients and tissue samples

One hundred and eleven NPC patients without distant metastasis (M0 stage) at the time of diagnosis who were treated by radical radiotherapy alone in the Affiliated Cancer Hospital of Central South University, China between Jan 2006 and Dec 2008 were recruited in this study. The radiotherapy was administered for a total dose of 60-70Gy (2Gy/fraction, 5 days a week). The neck received 60Gy for lymph node-negative cases and 70Gy for lymph node-positive cases. NPC tissue biopsies were obtained at the time of diagnosis before any therapy, fixed in 4% formalin and embedded in paraffin, and used for miR-23a qRT-PCR and immunohistochemistry of IL-8 and phospho-Stat3. We also acquired 16 cases of formalin-fixed and paraffin-embedded normal nasopharyngeal mucosa in the same period. On the basis of the 1978 WHO classification [39], all tumors were histopathologically diagnosed as poorly differentiated squamous cell carcinomas (WHO type III). The clinical stage of the patients was classified according to the 2008 NPC staging system of China [40].

The radiotherapy response was evaluated clinically for primary lesions based on nasopharyngeal fiberscope and magnetic resonance imaging (MRI) one month after the initiation of radiotherapy according to the following criteria. Radioresistant NPC patients were defined as ones with persistent disease (incomplete regression of primary tumor and/or neck lymphonodes) at > 3 months or with local recurrent disease at the nasopharynx and/or neck lymphonodes at ≤12 months after completion of radiotherapy. Radiosensitive NPC patients were defined as ones without the local residual lesions (complete regression) at > 3 months and without local recurrent disease at > 12 months after completion of radiotherapy. Based on the above criteria, one hundred and eleven NPC patients comprised 53 radioresistant and 58 radiosensitive ones.

The patients were followed up, and the follow-up period at the time of analysis was more than 72 months (average, 77.5 &plusmn; 11.8 months). Disease-free survival was calculated as the time from the completion of primary radiotherapy to the date of pathological diagnosis or clinical evidence of local failure and/or distant metastasis. Overall survival was defined as the time from the initiation of primary radiotherapy to the date of cancer-related death or when censured at the latest date if patients were still alive. The clinicopathologic parameters of the patients used in the present study are shown in Supplementary Table S1.

### QRT-PCR

Total RNA was extracted from cells with Trizol reagent (Invitrogen), or from the formalin-fixed and paraffin-embedded tissues with RecoverAll^TM^ total nucleic acid isolation kit (Ambion, Austin, TX, USA) according to the manufacturer’s instructions. qRT-PCR detection of miR-23a and IL-8 expression in NPC cells and tissues was performed as described previously [18]. The products were quantitated using 2^−^*^DDCt^* method against GAPDH or U6 for normalization. The primer sequences are listed in Supplementary Table S2.

### Transfection of miR-23a mimic and inhibitor into NPC cells

Cells were cultured with RPMI-1640 medium containing 10% FBS overnight, and then 50 nmol/L miR-23a mimic, miR-23a inhibitor and their respective negative control (RiboBio, Guangzhou, China) were transfected into cells using riboFect™ CP Transfection Kit (Ribobio) according to the manufacturer’s instruction, respectively. 24h after transfection, the experiments were performed on the transfected cells.

### Establishment of NPC cell lines with stable knockdown of IL-8

Psi-LVRU6GP-IL-8 shRNA and psi-LVRU6GP-scramble non-target shRNA, which established by GeneCopoeia (Rockville, MD, USA) and confirmed by sequencing, were transfected into CNE2-IR cells using Lipofectamine 2000 (Invitrogen), respectively. Cells were selected using puromycin for 2 weeks, and stable knockdown of IL-8 CNE2-IR cell lines and control cell lines were obtained. The targets for human IL-8 shRNA are show in Supplementary Figure S1.

### Clonogenic survival assay

A clonogenic survival assay was performed as described previously [30]. Briefly, cells were exposed to a range of radiation doses (1-8Gy), and 12d after irradiation surviving colonies were stained with 0.5% crystal violet and counted. The survival fraction was calculated as the numbers of colonies divided by the numbers of cells seeded times plating efficiency. Radiation dose-response curves were created by fitting the data to the linear quadratic equation *S* = *e*^−^*^α^*^D−^*^β^*^D^2^ using GraphPad Prism 5.0 (GraphPad Software Inc.), where *S* is the surviving fraction,α and β are inactivation constants, and *D* is the dose in Gy. The area under the curves (AUC) that represent the mean inactivation dose (MID) was also calculated using GraphPad Prism 5.0. The radiation protection factor (RPF) was calculated by dividing the MID of the test cells by the MID of control cells.

### *In vivo* tumor radioresponse assay

Nude male mice that were 4 weeks old were obtained from the Laboratory Animal Center of Central South University (Changsha, China). 5&times;10^6^ CNE2-IR cells were injected subcutaneously into the right flanks of 5-week-old nude mice. When the xenograft volumes reached approximately 50 mm^3^, the transplanted mice were randomly divided into 2 groups (*n* = 5 mice each), 5 nmol miR-23a agomir or control agomir (RiboBio) in 50&micro;l saline buffer was intratumorally injected into the tumor mass at multiple sites per mouse, and next day a 8 Gy dose of ionizing radiation was delivered to the tumor. 3 days after irradiation, 5nmol miR-23a agomir or control agomir was intratumorally injected into the tumor mass at multiple sites per mouse. 5 weeks after irradiation, the mice were killed by cervical dislocation, and their tumors were excised, weighted, and cut in half, with one half fixed embedded in paraffin for TUNEL and immunohistochemical staining, and the remaining half flash frozen in liquid nitrogen until use. Tumor volume (in mm^3^) was measured by caliper measurements performed every 3 to 4 days and calculated by using the modified ellipse formula (volume = length&times;width^2^/2).

### Flow cytometry analysis of cell cycle in response to irradiation

Flow cytometry analysis of cell cycle in response to irradiation was performed as previously described by us [30].

### Hoechst 33258 staining of apoptotic cells

Hoechst 33258 staining was performed to detect apoptotic cells after irradiation as previously described by us [32].

### Western blotting

Proteins were exacted from cells and tissues using RIPA buffer. An equal amount of protein in each sample was mixed with Laemmli buffer and subjected to sodium dodecyl sulfate-polyacrylamide gel electrophoresis (SDS-PAGE) separation, followed by blotting onto a polyvinylidene difluoride membrane(Millipore, Billerica, MA, USA). Blots were blocked with 5% nonfat dry milk or 3% BSA for 2 h at room temperature and then incubated with 1:1000 dilution of anti-IL-8 antibody (ab18672, Abcom, Cambridge, MA, USA), or 1:1000 dilution of anti-phospho-Stat3(Tyr705) antibody (#9131, CST, Beverly, MA, USA), or 1:1000 dilution of anti-Stat3 antibody (#4904, CST) overnight at 4C&deg;, followed by incubation with 1:3,000 dilution of horseradish peroxidase-conjugated secondary antibody for 1h at room temperature. The signal was visualized with an enhanced chemiluminescence detection reagent (Pierce, Minneapolis, MN, USA). β-Actin was detected simultaneously using 1:5000 dilution of monoclonal mouse anti-β-actin antibody (Sigma) as a loading control.

### *In situ* detection of apoptotic cells in the xenograft tumors

Terminal deoxynucleotidyl transferase(TdT)-mediated dUTP nick end labeling (TUNEL) was performed to detect apoptotic cells of formalin-fixed and paraffin-embedded tissue sections of xenograft tumors after irradiation with *In Situ* Cell Death Detection Kit (Roche, Basel, Switzerland, USA) according to the manufacturer’s instruction. Quantitative evaluation of apoptotic cells was done by examining the sections in ten random microscopic fields and counting the number of TUNEL-positive cancer cells among 1000 carcinoma cells under the light microscope. The apoptotic index was expressed as positive cells per 100 cancer cells.

### Immunohistochemistry

Immunohistochemical staining of IL-8, phospho-Stat3 (Tyr705) and γH2AX (phospho-S139), a marker for DNA double-strand breaks, were performed on formalin-fixed and paraffin-embedded tissue sections as previously described. Briefly, after antigen retrieval tissue sections were incubated with anti-IL-8 antibody (ab18672, Abcom), phospho-Stat3 (Tyr705) antibody (1:400 dilution) (#9145; CST) or γH2AX antibody (ab2893, Abcom) overnight at 4&deg;C, and then were incubated with biotinylated secondary antibody followed by avidin-biotin peroxidase complex (DAKO, Glostrup, Denmark). Finally, tissue sections were incubated with 3′, 3′-diaminobenzidine (Sigma, St Louis, MO) and counterstained with hematoxylin. In negative controls, primary antibodies were omitted. The immunoreactions of IL-8 and phospho-Stat3 were evaluated independently by two pathologists as described previously. Staining intensity was categorized: absent staining as 0, weak as 1, moderate as 2, and strong as 3. The percentage of stained cells was categorized as no staining = 0, < 30% of stained cells = 1, 30∼60% = 2, and > 60% = 3. The staining score (ranging from 0-6) for each tissue was calculated by adding the area score and the intensity score. A combined staining score of≤3 was considered to be low expression; and a score of > 3 was considered to be high expression. Quantitative evaluation of DNA damaged cells was done by examining the sections in ten random microscopic fields and counting the number of γH2AX positive nuclei among 1000 carcinoma cells under the light microscope. The rate of DNA damaged cells was expressed as positive cells per 100 cancer cells.

### Dual luciferase reporter assay

5&times;10^5^cells were plated into 60 mm culture dish and incubated with RPMI-1640 medium containing 10% FBS for 12h. Cells were transiently cotransfected with 0.5 &micro;g of a reporter plasmid containing human STAT3 response element (pSTAT3-TA-luc) (Beyotime, Nanjin, China) and 0.5 &micro;g of pRL-TK plasmid (Promega, Madison, WI, USA) using lipofectamine 2000 in serum-free medium. Cotransfection of pGL6-TA without STAT3 response element (Beyotime) and pRL-TK plasmid into cells served as a control. Cells were harvested 48h after transfection, and both firefly luciferase and renilla luciferase activities were measured with the Dual-luciferase reporter assay system (Promega) according to the manufacturer’s instruction, and transcriptional activity of Stat3 was estimated using a luminometer.

### Immunofluorescent staining

Immunofluorescent staining was performed to detected the nuclear translocation of phospho-Stat3 in the NPC cells using anti-phospho-Stat3 (Tyr705) antibody (#9145, CST) and secondary antibody conjugated with Alexa Fluor 594 (Vector Laboratories) as previously described by us [41].

### Antibody neutralization of secretory IL-8

Cells were cultured with RPMI-1640 medium containing 2% FBS and 2.5 &micro;g/mL mouse anti-human IL-8 antibody (ab18672, Abcom) or 2.5 &micro;g/mL mouse control IgG1 (ab188776, Abcom) for 24h, and then cells were subjected to further analysis.

### Stimulation of cells by IL-8

Cells were cultured with RPMI-1640 medium containing 10% FBS for 24h, and incubated in a serum-free in medium for an additional 12h, and then 1.5 or 4.5 ng/mL of recombinant human IL-8 (Life technologies, Carlsbad, CA) were added into the medium. 12h after stimulation, cells were subjected to further analysis.

### Transient transfection of Stat3 expression vector

Cells were plated in 6-well culture plates, and incubated with RPMI-1640 medium containing 10% FBS 12h before transfection. The transfection of recombinant plasmid pReceiver-M13-Stat3 or control vector pReceiver-M13 (GeneCopoeia) (the final concentration: 4 μg/mL) into cells was performed using Lipofectamine 2000. 48h after transfection, cells were subjected to further analysis.

### Statistical analysis

All experiments were carried out at least 3 times. Data were presented as the mean &plusmn; standard deviation (SD). Statistical analysis was conducted using SPSS 20.0 software. For comparisons between two groups, a Student t test or chi-square test was used. Survival curves were obtained by using Kaplan-Meier method, and comparisons were made by using log-rank test. Univariate and multivariate survival analyses were conducted on all parameters by using Cox proportional hazards regression model. The Spearman rank correlation coefficient was used to determine the correlation between two parameters. P values less than 0.05 were considered to be statistically significant.

### Ethics statement

This study was approved by the ethics committee of Xiangya School of Medicine, Central South University, China. Written informed consent was obtained from all participants in the study. All animal experiments were undertaken in accordance with the Guide for the Care and Use of Laboratory Animals of Central South University, with the approval of the Scientific Investigation Board of Central South University.

## SUPPLEMENTARY MATERIAL FIGURE AND TABLES


