# Supplementary material for: MiR-23a sensitizes nasopharyngeal carcinoma to irradiation by targeting IL-8/Stat3 pathway
Source: Oncotarget. 2015 Jun 7;6(29):28341–56. doi: 10.18632/oncotarget.5117 (PMC4695064; doi:10.18632/oncotarget.5117)
Supplement: Supplementary file 1 [file oncotarget-06-28341-s001.pdf]

MiR-23a sensitizes nasopharyngeal carcinoma to irradiation by targeting IL-8/Stat3 pathway

Supplementary Material

No.	Vector Name	Length	Target Sequence	Chosen
IL8 shRNA1	HSH009678-5-LVRU6GP(CS9OS397270)	21	gcactccataaggcacaaact	√
IL8 shRNA2	HSH009678-6-LVRU6GP(CS9OS397271)	21	gcataaagacatactccaaac	√
IL8 shRNA3	HSH009678-7-LVRU6GP(CS9OS397272)	21	gcacgggagaatatacaaata	
IL8 shRNA4	HSH009678-8-LVRU6GP(CS9OS397273)	21	ggatccacaagtccttgttcc	

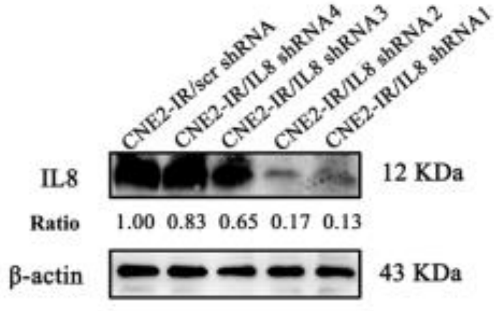

Supplementary Figure S1. The targets for human IL-8 shRNA vector and their efficacy of IL-8 knockdown. (top) the targets for human IL-8 shRNA; (bottom) a representative result of Western blotting shows that the expression levels of IL-8 in the CNE2-IR cells transiently transfected with the psi-LVRU6GP-IL-8 shRNA vector expressing IL-8 shRNA1, shRNA2, shRNA3 or shRNA4. Knockdown efficacy of IL-8 shRNA1 and shRNA2 was better than that of the other two IL-8 shRNAs, thus IL-8 shRNA1 and shRNA2 vectors were selected for establishing stably transfected CNE2-IR cell lines.

Supplementary Table S1. The clinicopathological parameters of 111 patients with nasopharygeal carcinoma

Variable	No. of patients	%
**Gender**		
Male	88	79.28
Female	23	20.72
**Age**		
≥46	52	46.85
<46	59	53.15
**Primary tumor(T) stage**		
T1-2	62	55.86
T3-4	49	44.14
**Lymph node(N) metastasis**		
N0	23	20.72
N1-3	88	79.28
**Clinical stage**		
I-II	25	22.52
III-IVa	86	77.48
**Radiotherapeutic response**		
Radiosensitive	58	52.25
Radioresistant	53	47.74

Supplementary Table S2. QRT-PCR primers for amplification of miR-23a and IL-8 synthesized by Ribobio

No.	Name	RT primer	Primer sequence
1	miR-23a	Cat.#, SSD809230260	F: Cat.#, SSD809230952
			R: Cat.#, SSD0892261711
2	U6	Cat.#, SSD0904071008	F: Cat.#,SSD0904071006
			R: Cat.#,SSD0904071007
3	IL-8	Oligo dT	F: 5’-TGGCAGCCTTCCTGATTT-3’
			R: 5’-AACCCTCTGCACCCAGTT-3’
4	GAPDH	Oligo dT	F: 5’-TGACTTCAACAGCGACACCCA-3’
			R: 5’-CACCCTGTTGCTGTAGCCAAA-3’
